# Cutaneous Injection of Resiniferatoxin Completely Alleviates and Prevents Nerve-Injury-Induced Neuropathic Pain

**DOI:** 10.3390/cells11244049

**Published:** 2022-12-14

**Authors:** Hayate Javed, Aishwarya Mary Johnson, Anil Kumar Challagandla, Bright Starling Emerald, Safa Shehab

**Affiliations:** Department of Anatomy, College of Medicine and Health Sciences, United Arab Emirates University, Al-Ain P.O. Box 15551, United Arab Emirates

**Keywords:** neuropathic pain, TRPV1, resiniferatoxin, cutaneous injection, spinal cord, DRG, nerve injury

## Abstract

Fifth lumbar (L5) nerve injury in rodent produces neuropathic manifestations in the corresponding hind paw. The aim of this study was to investigate the effect of cutaneous injection of resiniferatoxin (RTX), a TRPV1 receptor agonist, in the rat’s hind paw on the neuropathic pain induced by L5 nerve injury. The results showed that intraplantar injection of RTX (0.002%, 100 µL) (1) completely reversed the development of chronic thermal and mechanical hypersensitivity; (2) completely prevented the development of nerve-injury-induced thermal and mechanical hypersensitivity when applied one week earlier; (3) caused downregulation of nociceptive pain markers, including TRPV1, IB4 and CGRP, and upregulation of VIP in the ipsilateral dorsal horn of spinal cord and dorsal root ganglion (DRG) immunohistochemically and a significant reduction in the expression of TRPV1 mRNA and protein in the ipsilateral DRG using Western blot and qRT-PCR techniques; (4) caused downregulation of PGP 9.5- and CGRP-immunoreactivity in the injected skin; (5) produced significant suppression of c-fos expression, as a neuronal activity marker, in the spinal neurons in response to a second intraplantar RTX injection two weeks later. This work identifies the ability of cutaneous injection of RTX to completely alleviate and prevent the development of different types of neuropathic pain in animals and humans.

## 1. Introduction

Neuropathic pain is a chronic debilitating condition resulting from lesions or diseases affecting the somatosensory nervous system [1]. Accordingly, four main animal models were developed to investigate the mechanism and management of neuropathic pain comprising nerve injury [2]. For example, spinal nerve ligation (SNL) is the most commonly used and a well-established model in which the injury of L5 or L5 and L6 spinal nerves in rats would lead to the development of allodynia and hyperalgesia in the ipsilateral hind paw in rats [3].

Despite advances in understanding the complex neurobiology of pain, treating neuropathic pain is still a challenge. At present, the management of neuropathic pain is inadequate, with less than half of the patients reporting satisfactory (50% or better) pain relief after treatment [4]. In addition, there is no method to prevent it. There remains, therefore, a pressing unmet need for more effective treatments for patients with this neuropathic debilitating chronic condition. Therefore, the development of novel therapeutic interventions will be critical [5]. Pharmacological treatment with various classes of drugs is typically the first line of management of neuropathic pain. However, these drugs are normally used only for short periods and not exceeding 4–8 weeks. To achieve sufficient pain relief, high doses of the medications are often needed. Unfortunately, this can invoke many undesirable and limiting side effects [6]. The problem is compounded in up to 45% of cases where patients are treated with two or more drugs [7]. Regardless of the efficacy of drugs with different mechanisms, the effects are limited and frequently accompanied by side effects, and the majority of patients will not have enough pain relief at doses they can tolerate [5].

However, one of the promising recent approaches to new peripheral analgesics is the local application of capsaicin (the ingredient of hot chili peppers) or its ultrapotent analogue resiniferatoxin (RTX). Capsaicin and RTX act through their specific heat receptor, known as transient receptor potential vanilloid type 1 (TRPV1) [8,9,10], which is widely known for its expression on the unmyelinated C-fibers and small–medium DRG neurons [11,12,13,14,15,16]. The topical application of capsaicin to the skin initially induces a burning pain sensation, followed by prolonged attenuation of the pre-existing pain from the same region [17,18,19]. Therefore, capsaicin and RTX have been widely used as a tool for treating pain in both clinical and preclinical studies [20,21,22].

When administered directly onto peripheral nerves, capsaicin and RTX were shown to produce selective thermal and chemical analgesia accompanied by the abolition of neurogenic inflammation in the skin area supplied by the treated nerve [23]. Cutaneous injection of RTX attenuated thermal nociception [24,25], blocked the inflammation-induced hyperalgesia [26,27], and produced long-lasting analgesia in a rat model of pain associated with burn injury [28]. In addition, cutaneous injection of RTX in rats also showed behavioral recovery through regeneration of TRPV1-expressing Aδ and C-fibers, which were stimulated by infrared laser to study the fiber-type specific behavior [13]. Furthermore, injections of the RTX in the rat DRG prevented tactile allodynia induced by photochemical injury of the sciatic nerve [29], or in the trigeminal ganglion of rat and monkey, caused the elimination of hyperalgesia and neurogenic inflammation [25,30].

Recently, we have shown that perineural application of either capsaicin or RTX on the uninjured adjacent L4 nerve produced significant alleviation of the thermal and mechanical hypersensitivity caused by L5 nerve injury in rats [11]. Moreover, to completely alleviate the thermal and mechanical hypersensitivity in this model of neuropathic pain, treatment of both L3 and L4 nerves with RTX (0.002%) was required. In this study, we aimed to determine whether intraplantar injection of RTX in the hind paw of rats could (i) produce complete analgesic effects (ii) and completely prevent the development of the thermal and mechanical hypersensitivity in the ipsilateral hind paw induced by L5 nerve injury. In the next step, we investigated the mechanism of the analgesic effect of intraplantar RTX injection. To achieve that, we determined the neurochemical changes in the skin, DRG and spinal cord of RTX-treated rats. In addition, we determined the expression of mRNA and the protein level of TRPV1 in the corresponding DRGs.

## 2. Materials and Methods

### 2.1. Animals

Male Wistar rats were bred and maintained at the animal research facility of the College of Medicine and Health Sciences (CMHS) at the United Arab Emirates (UAE) University. Animals were maintained on a 12-hr dark/light cycle with food and water ad libitum. All experimental procedures were approved by the Animal Ethics Committee of the CMHS, UAE University (Approval Code: ERA-2020-7222, Approval Date: 19 January 2021), and were performed in accordance with the guidelines of the European Communities Council Directive of 24 November 1986 (86/609/EEC).

### 2.2. L5 Nerve-Injury-Induced Neuropathic Pain Model, Intraplantar Injection of RTX/Vehicle and Behavioral Testing

#### 2.2.1. Surgery Rats

The rats (weighing 240–255 g) underwent L5 spinal nerve ligation, as reported previously [3,11,31]. Briefly, rats were anesthetized with 2–2.5% isoflurane in an air mixture administered through a nose cone. The skin of the back was incised longitudinally and the transverse processes of the sixth lumbar vertebrae were excised. The left L5 nerve was ligated with a 6/0 silk suture and sectioned distally. Subsequently, the muscles and the skin were sutured in layers and rats were given appropriate postoperative care.

#### 2.2.2. Behavioral Testing

Rats (*n* = 14) were acclimatized in the thermal and mechanical behavioral testing chambers located on a glass platform or metal mesh for thermal or mechanical hyperalgesia, respectively, for at least 2 h every day for 3 days and then the baseline values of the paw withdrawal latency were measured in response to thermal and mechanical stimuli. Thereafter, rats underwent L5 nerve injury, as described above. After 3 and 7 days of L5 nerve injury, rats were tested in response to thermal and mechanical stimuli to confirm the hyperalgesia in rats. To treat the L5 nerve-injury-induced hyperalgesia, on day 7, rats were injected with 100 µL of RTX (Sigma, St. Louis, MO, USA) 0.002% (2 µg) or vehicle (10% Tween 80, 10% EtOH, 80% normal saline) in the plantar skin of the left hind paw (n = 7 each group). The intraplantar injection of RTX/vehicle was carried out under very brief isoflurane anesthesia.

In another protocol, animals were tested for baseline values in response to thermal and mechanical stimuli (*n* = 14). Subsequently, rats were injected with 100 µL of RTX (0.002%) or vehicle in the plantar skin of the left hind paw. After RTX/vehicle injections, rats were tested again on days 3 and 7 in response to thermal and mechanical stimuli. On day 7, rats underwent left L5 nerve injury as described above. Later, we assessed the effect of pre-injected RTX/vehicle on the paw withdrawal latency in response to thermal and mechanical stimuli 3–28 days after L5 nerve injury. The different groups of animals and their respective treatments are mentioned in the Table 1.

The behavioral tests were performed between 8:00 and 16:00 h. The thermal and mechanical thresholds of paw withdrawal were assessed using a plantar and dynamic plantar aesthesiometer, respectively (Ugo Basile, Gemonio (Va), Italy). The withdrawal latency for thermal hypersensitivity was recorded by a digital timer connected to a mobile radiant heat source located underneath the glass focused on one hind paw. The infrared stimulus was set at 70 a.u. with a cut-off latency of 20 s to prevent tissue damage. Mechanical hypersensitivity was measured with a movable force actuator positioned below the plantar surface of the animal and the desired force and force speed was applied at 2.5 g/s. A Von Frey-type 0.5-mm filament exerted force incrementally until the animal briskly withdrew the hind paw. At each paw withdrawal, a digital recorder connected to the movable force actuator recorded the latency time and the actual force applied during the paw withdrawal reflex. The investigator who performed the behavioral tests was blinded to the types of treatments and groups.

### 2.3. Immunohistochemistry

RTX (0.002%) or vehicle 100 µL was injected in the plantar skin of left hind paw, and after 14 days, the animals were perfused. Briefly, animals were anesthetized with sevoflurane and perfused through the ascending aorta with 4% paraformaldehyde in 0.1 M phosphate buffer (pH 7.4). The lumbar spinal segments L3, L4 and L5 were removed and postfixed in the same fixative for 3–4 h. The fourth ipsilateral and contralateral DRGs were postfixed for 1 h. Moreover, the skin of the injected area of the hind paw (ipsilateral and contralateral) was also dissected out and postfixed for 1 h. All samples were stored in 30% sucrose in phosphate buffer overnight at 4 °C.

#### 2.3.1. Spinal Cord and DRG Staining

Cryostat transverse sections (50 μm) of L3, L4 and L5 spinal segments were prepared and treated with 50% ethanol to increase antibody penetration [32]. Serial sections of L4 DRG (15 μm thick) were also collected from the ipsilateral and contralateral sides on gelatin-coated slides. The sections were incubated overnight with guinea pig anti-TRPV1, rabbit anti-VIP and goat anti-IB4. For IB4 staining, the sections were preincubated with IB4 lectin (Vector Laboratories, Burlingame, CA; 1 µg/mL) for 1 h. In another set of staining, sections were incubated with guinea pig anti-TRPV1, rabbit anti-VIP and sheep anti-CGRP. Furthermore, spinal cord sections were also incubated with guinea pig anti-TRPV1 and rabbit anti-SP for double immunofluorescence labelling. After rinsing with phosphate-buffered saline (PBS; pH 7.4), the sections were incubated for 1 h in species-specific secondary antibodies that were raised in donkeys (anti-guinea pig conjugated to Alexa 488 diluted to a concentration of 1:200, anti-rabbit conjugated to Rhodamine Red and anti-goat conjugated to Cyanine 5 [Cy5] diluted to a concentration of 1:100 [Jackson Immuno Research, West Grove, PA, USA].

In addition, rats injected with RTX (0.002%) or vehicle in the left hind paw were perfused either 40 h or 14 days after injection to observe and quantify the number of VIP-positive (VIP+) and TRPV1-positive (TRPV1+) neurons and their colocalization in the DRG sections. The DRG sections were incubated overnight with a mixture of primary antibodies, including guinea pig anti-TRPV1 and rabbit anti-VIP. After rinsing, the sections were incubated for 1 h in secondary antibodies (anti-guinea pig conjugated to Alexa 488 and anti-rabbit conjugated to Rhodamine Red). For nuclear staining, sections were then counterstained with 4′, 6-diamidino-2-phenylindole, di-hydrochloride (DAPI, Molecular Probes, Life Technologies, Carlsbad, CA, USA).

All sections were mounted in antifade medium immuno-mount (Thermo Fisher Scientific, Runcom, Cheshire, UK).

#### 2.3.2. c-Fos Staining in the Spinal Cord (ABC Method)

In order to assess the effects of intraplantar injection of RTX on the pain marker c-fos, rats were injected with RTX 0.002% or vehicle in the skin of the left hind paw. After 14 days of injections, rats were again injected with only RTX 0.002% in the left hind paw of both groups (RTX- and vehicle-injected), and 90 min following injections, the rats were perfused as described above. The spinal segments L3, L4 and L5 were dissected out and postfixed for 3–4 h and, subsequently, transferred into 30% sucrose overnight at 4 °C. Cryostat transverse sections (50 μm) of L3, L4 and L5 spinal segments were prepared and processed for immunohistochemistry. The sections were incubated overnight with rabbit anti-c-fos (1:5000) at room temperature. After rinsing with PBS, the sections were incubated with biotinylated anti-rabbit secondary antibody (1:500; Jackson Immuno Research, West Grove, PA, USA) for an hour, followed by extravidin peroxidase conjugate for 1 h (1:1000, Sigma-Aldrich, St. Louis, MO, USA). Finally, the sections were incubated for 4–5 min in a solution of 3,3’-diaminobenzidine (DAB) solution (25 mg/50 mL of phosphate buffer, pH 7.4 with 7.5 µL hydrogen peroxide [30%] and 1 mL nickel chloride [3%] added to it). All sections were mounted on gelatin-coated slides and allowed to air-dry overnight. They were then washed, dehydrated in graded alcohol, cleared in xylene, coverslipped and examined under a light microscope. The quantification of c-fos labelled nuclei was carried out in the medial half area of the dorsal horn of the L3, L4 and L5 spinal segments from each group. Images from eight randomly selected sections were captured at 10× objective and the number of labelled nuclei was manually counted using Image J software (v1.53k).

#### 2.3.3. Skin Staining

Fourteen days after the intraplantar injection of RTX (0.002%) or vehicle (100 µL), the skin of the injected area of the left hind paw and control right hind paw were collected after perfusion with 4% paraformaldehyde as described above. The skin was sectioned transversally (15 µm) by cryostat and collected on the gelatin-coated slides. The sections were incubated overnight with rabbit anti-PGP 9.5 and sheep anti-CGRP. After rinsing with PBS, the sections were incubated for 1 h in species-specific secondary antibodies that were raised in donkeys (anti-rabbit conjugated to Rhodamine Red diluted to a concentration of 1:100 and anti-goat conjugated to Alexa 488 diluted to a concentration of 1:200). Sections were mounted in antifade medium immuno-mount (Thermo Fisher Scientific, Runcom, Cheshire, UK).

Details of the primary antibodies used and their source and dilutions are given in Table 2.

### 2.4. Quantitative Real-Time Polymerase Chain Reaction

The mRNA expression level of TRPV1 was quantified after 14 days of intraplantar injection of RTX (0.002%) or vehicle in the left hind paw. Total RNA was extracted from left L3 and L4 DRGs from RTX and vehicle-treated rats, as well as right control L3 and L4 DRGs (*n* = 3–5). The qRT-PCR analysis was performed as we published previously [11,33]. Briefly, total RNA was extracted using TRIzol™ Reagent (Thermo Fisher Scientific, California, USA) as per the manufacturer’s instructions. The total RNA (1 μg) was converted to cDNA using Applied Biosystems high-capacity cDNA reverse transcription kit (Applied Biosystems, Bedford, MA, USA). PCR reactions were carried out in a volume of 20 μL of Luna^®^ Universal qPCR Master Mix (New England BioLabs, Ipswich MA, USA) with 200 ng of cDNA and 0.25 μM of each primer, using QuantStudio™ 3 Real-Time PCR System (Applied Biosystems). The relative gene expression was calculated using the comparative Ct method [34]. Furthermore, 18S RNA was used as the internal control.

The primers used were as follows: Trpv1 F5′ CATGGGTG AGACCGTCAA CA 3′; Trpv1 R5′ AGGCCTTCCTCATGCACTTC 3′; 18S F5′ AGTCCCTGCCCTTTGTACACA 3′; 18S R5′ GATCCGAGGG CCTCACTAAAC 3′.

### 2.5. Western Blot

The protein expression level of TRPV1 was measured by Western blotting after 14 days of RTX 0.002% or vehicle injection in the left hind paw of rats. The left L3 and L4 DRGs from RTX and vehicle-treated rats as well as right L3 and L4 control DRGs were homogenized in RIPA buffer with protease and phosphatase inhibitors as described previously [11,33]. The tissue lysates were then centrifuged at 15,000 rpm for 20 min. The supernatant was isolated, and the protein concentration was quantified using the Pierce BCA protein assay kit (Thermo Fisher Scientific, Rockford, IL) following the manufacturer’s instructions. Subsequently, equal amounts of protein (30 μg) were loaded and separated using 4–20% SDS–polyacrylamide gel electrophoresis. The proteins were then transferred onto a PVDF membrane and incubated overnight at 4 °C with a specific primary antibody rabbit polyclonal against TRPV1 (1:1000) [CAT No. BML SA-564, ENZO Life Sciences, Lausen, Switzerland]. The membrane was washed and then incubated with horseradish peroxidase-conjugated secondary antirabbit antibody. The protein recognized by the antibody was visualized using an enhanced chemiluminescence Pico kit (Thermo Fisher Scientific, Rockford, ILz, USA). As a loading control, the blots were stripped and re-probed for β-actin (1:5000; monoclonal mouse, Millipore, MA, USA). The intensity of the bands was measured using densitometry and quantified using Image J software (NIH, Bethesda, MD, USA).

### 2.6. Imaging and Data Analysis

The sections were examined with a Nikon fluorescent microscope (Nikon, Tokyo, Japan) equipped with appropriate filters to reveal Alexa 488 (green fluorescent) and Rhodamine Red (red fluorescent) or a Nikon C1 laser scanning confocal microscope to reveal Alexa 488, Rhodamine Red and Cy5 (blue fluorescent) labelling. Representative digital images were captured using either a Nikon DS-Ri2 camera or a Nikon C1 confocal microscope. The resulting files were used to generate figures in Adobe Photoshop software CS6 (San Jose, CA, USA) where photomicrographs were adjusted for contrast and brightness.

### 2.7. Dorsal Root Ganglion

The quantification of neuropathic pain manifestations related to nociceptive markers was carried out in the ipsilateral and contralateral DRGs from animals injected with 0.002% RTX or vehicle in the left hind paw. From the serially sectioned DRGs, counting was performed in every ninth serial section (5 sections per DRG). All DRG sections were counterstained with DAPI to determine the percentage of neurons labelled with TRPV1, VIP, IB4 and CGRP in relation to the total number of neurons in each DRG. The colocalization of these markers in the individual cells was confirmed by the Neurolucida software (MBF Biosciences, Williston, VT, USA). The percentage of individual markers was calculated by dividing the total number of DAPI-positive (DAPI+) neurons that contain other markers by the total number of DAPI+ neurons and multiplying the result by 100. The percentage of double-labelled neurons was also measured.

### 2.8. Antibody Characterization

The TRPV1 antibody selectively and specifically recognizes TRPV1 of rat and mouse origin (manufacturer’s technical information). We have previously characterized this antibody [11], and the pretreatment of guinea pig anti-TRPV1 antibody with TRPV1 peptide (Neuromics, MN, USA) at a concentration of 10^−5^ M completely abolished the positive staining compared with sections incubated with only TRPV1 antibody. In addition, this TRPV1 antibody showed negative labelling in the DRG of TRPV1−/− mice [35]. The radioimmunoassay data revealed that CGRP antibody recognizes canine, rat and mouse α-CGRP (Peninsula datasheet). Rabbit anti-CGRP and sheep anti-CGRP antibodies incubated with CGRP peptide (PolyPeptide Group, San Diego, CA, USA) at the concentration of 10^−5^ M showed complete abolishment of positive labelling compared with sections incubated with the CGRP antibody alone [11]. The pretreatment of rabbit anti-VIP antibody with VIP (10^−6^ M) and rabbit anti-SP antibody with SP (10^−5^ M) completely abolished the positive staining [31,36]. Goat anti-IB4 antibody was produced against Griffonia simplicifolia lectin I as an immunogen. This antibody was purified using affinity chromatography on lectin-specific columns and yielded identical recognition when tested through immunodiffusion (manufacturer’s technical information). The spinal cord sections of control rats incubated with IB4 antibody showed no immunoreactivity. Furthermore, the specificity of the IB4 antibody was shown by the presence of immunoreactivity in a specific area of the spinal cord (lamina II), while other areas showed no positive labelling [31]. In addition, sections that were not incubated with IB4 revealed no positive labelling.

The rabbit anti-PGP 9.5 antibody was produced by repetitive injection of purified whole human PGP 9.5 in Freund’s adjuvant into rabbits. All mammalian species’ PGP 9.5 proteins are cross-reactive with the antibody (manufacturer’s technical information). This antibody recognizes a band at 38 kDa from human and rat skin by Western blotting. The pretreatment of rabbit anti-PGP 9.5 with purified PGP 9.5 completely abolished the positive staining [37]. The rabbit anti-c-fos antibody recognizes a band at 56 kDa, and uncharacterized bands may be observed in some lysate(s). This antibody specifically reacts with human and rat tissues and is predicted to react with other species based on 100% sequence homology (manufacturer’s technical information). Furthermore, no immunoreactivity was detected in sections incubated with non-immune rabbit or goat serum.

### 2.9. Statistical Analysis

The behavioral data are expressed as mean of thermal (s) and mechanical (g) paw withdrawal latency values ± standard error of mean (SEM) and were analyzed with Graph Pad (InStat software, La Jolla, CA, USA) using a two-way analysis of variance (ANOVA) followed by Bonferroni’s post hoc test to determine the statistical significance between the means of various groups. The neuronal count data for nociceptive markers (TRPV1, VIP, IB4 and CGRP) are expressed as percent mean of neurons positively labelled with each of the above markers with respect to the total number of DAPI-labelled neurons ± SEM and analyzed using one-way ANOVA followed by Bonferroni’s post hoc test. Moreover, qRT-PCR and Western blotting data were also statistically analyzed by one-way ANOVA followed by Bonferroni’s post hoc test. Results were considered statistically significant at *p* < 0.05.

## 3. Results

### 3.1. Behavioural and Analgesic Effects of Plantar Injection RTX in Normal Rats

**Figure 1:**
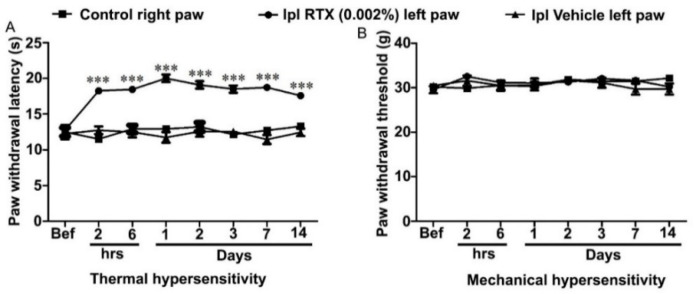
Thermal and mechanical paw withdrawal latency was measured before and after RTX (0.002%, 100 µL) or vehicle injection in the skin of left hind paw of rats. Thermal paw withdrawal latency of RTX-injected left hind paw was significantly (*** *p* < 0.001) increased at different time points compared to vehicle-injected left hind paw or right control paw (**A**). In contrast, no significant difference was observed in the mechanical paw withdrawal latency of RTX-injected left hind paw compared to vehicle-injected left hind paw or right control paw (**B**) [(*** RTX vs. vehicle/right control) *n* = 7 each group].

In a preliminary experiment, we tested the effects of RTX in normal rats. Rats were injected with RTX (0.002%, 100 µL) or an equal volume of vehicle in the plantar skin of the left hind paw. The animals appeared drowsy and sedated, which was very likely due to the general anesthesia, and showed paw flicking within the first 5 min following the injection. After 15 min of injection, there was no paw flicking or guarding but subtle amounts of edema and redness were observed. The animals’ mobility was reduced; however, normal movement was seen 2 h after the RTX injection. Vehicle-injected rats also showed paw flicking occasionally in the first 5 min with no paw guarding. However, a slight paw edema was noticed without redness. To assess the analgesic effects of the RTX injection, the animals were tested for thermal and mechanical sensations at different time points; 2 h, 6 h, 1,2, 3, 7 and 14 days after the injection. The results showed a significant (*p* < 0.001) increase in thermal paw withdrawal latency (almost reaching the cut-off level) in the RTX-injected left hind paw compared to the vehicle-injected left hind paw, as well as the right control hind paw withdrawal latencies (Figure 1A). However, no significant changes in the mechanical paw withdrawal latencies were observed among the RTX or vehicle-injected left paw and control right paw (Figure 1B).

### 3.2. Intraplantar Injection of RTX Completely Alleviates Thermal and Mechanical Hypersensitivity Caused by L5 Nerve Injury 

**Figure 2:**
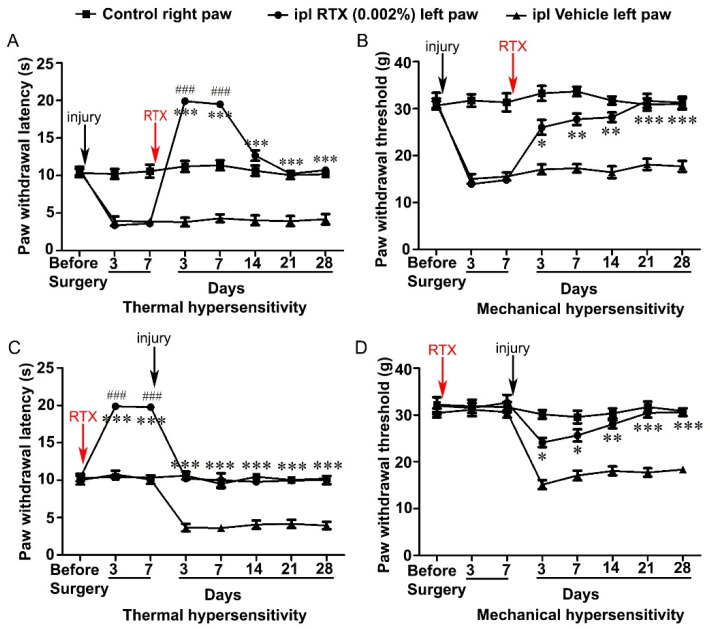
Analgesic effects of the intraplantar injection of RTX (0.002%) for the treatment (**A**,**B**) and prevention (**C**,**D**) of thermal and mechanical hypersensitivity induced by L5 nerve injury. RTX treatment after 3 and 7 days abolished L5 injury-induced thermal hypersensitivity and produced analgesic effects as the left foot became less sensitive to heat stimuli compared with right control foot (**A**). Interestingly, after 2–4 weeks, RTX-injected rats showed significantly increased thermal paw withdrawal latency compared to vehicle-injected rats but not with the right control paw latency (**A**). The mechanical hypersensitivity in RTX-injected animals was significantly reduced after 3–7 days compared with vehicle-injected rats, and the mechanical paw withdrawal latency returned to the basal level after 14 days compared with right control paw latency (**B**). Pretreatment of RTX showed a significant increase in the thermal paw withdrawal latency after 3 and 7 days compared to right control paw latency (**C**). In contrast, no significant difference was observed in mechanical paw withdrawal latency following RTX treatment (**D**). After 7 days of RTX treatment, the L5 nerve was injured to induce neuropathic manifestations. The results showed a significant increase in thermal and mechanical paw withdrawal latency in RTX-injected rats compared to vehicle-injected rats, but no significant difference was observed between left RTX-injected and right control paw withdrawal latencies (**C**,**D**). [(* RTX vs. vehicle; # RTX vs. right control) * *p* < 0.05; ** *p* < 0.01; *** *p* < 0.001, ### *p* < 0.001; *n* = 7 each group].

The rats were tested before the surgery to determine the baseline of thermal and mechanical paw withdrawal latencies, and then, neuropathic pain was induced by left L5 nerve injury. After 3 and 7 days, all animals showed significantly (*p* < 0.001) reduced left paw withdrawal latency compared to right paw withdrawal latency in response to thermal and mechanical stimuli, indicating that animals developed hypersensitivity due to L5 nerve injury. In order to determine the alleviation of the L5 nerve-injury-induced hypersensitivity, the animals were divided into two groups and they were injected either with RTX (0.002%, 100 µL) or the same volume of the vehicle in the plantar skin of the left hind paw. Subsequently, the animals were tested for thermal and mechanical hypersensitivity 3, 7, 14, 21 and 28 days after the RTX/vehicle injection. The results showed that the left paw thermal withdrawal latency of RTX-injected rats was significantly (*p* < 0.001) higher (reaching almost to the cut-off level) after 3 and 7 days of RTX treatment compared with the withdrawal latency of the left vehicle-injected and right control paw. These results showed that intraplantar injection of RTX not only abolished the thermal hypersensitivity but produced a dramatic analgesic effect and significantly less heat sensation compared with the right control paw (Figure 2A). Interestingly, the thermal paw withdrawal latency of the RTX-injected left paw was decreased after 14–28 days and the difference became insignificant compared with withdrawal latency of the right paw. However, it was still significantly more compared to the vehicle-injected left paw withdrawal latency. This indicates that thermal hypersensitivity was completely abolished and heat sensation returned to its basal level after 14–28 days. In the case of mechanical hypersensitivity, the withdrawal latency of the RTX-injected left paw was found to be significantly higher (*p* < 0.001) than the withdrawal latency of the vehicle-injected left paw (after 3–28 days of RTX injection). However, no significant difference was observed in the withdrawal latencies of the RTX-injected left paw and the right control paw after 3–28 days of RTX injection (Figure 2B).

### 3.3. Intraplantar Injection of RTX Completely Prevents the Development of Thermal and Mechanical Hypersensitivity Caused by L5 Nerve Injury 

In the next experiment, we investigated the effect of the pretreatment of RTX on the development of L5 nerve injury-induced thermal and mechanical hypersensitivity. Animals were first tested for the baseline measurements, and then, 0.002% RTX (100 µL) or the same volume of vehicle was injected in the plantar skin of the left hind paw. Consequently, animals were tested for thermal and mechanical hypersensitivity at 3- and 7-days post treatment. The results showed that the RTX-injected left paw thermal withdrawal latency was significantly (*p* < 0.001) increased (reaching almost to the cut-off level) compared to vehicle-injected and right control paw withdrawal latencies. In contrast, no significant changes in mechanical paw withdrawal latency were observed between the left paw of RTX or vehicle-injected or right control paw. After 7 days of RTX/vehicle treatment, the animals underwent left L5 nerve injury and again were tested for thermal and mechanical hypersensitivity after 3, 7, 14, 21 and 28 days. We observed that RTX-injected left paw withdrawal latency in response to thermal stimuli reached the right control paw withdrawal latency after 3–28 days of L5 nerve injury (Figure 2C). Furthermore, paw withdrawal latency in response to mechanical stimuli of RTX-injected left paw was significantly increased compared to vehicle-injected left paw 3–28 days after L5 nerve injury with no significant difference observed between RTX-injected left paw and control right paw mechanical withdrawal latencies (Figure 2D). These results indicated that intraplantar injection of RTX completely prevented the nerve-injury-induced thermal and mechanical hypersensitivities.

### 3.4. Effects of Intraplantar Injection of RTX on the Immunoreactivity of TRPV1, IB4, CGRP, SP and VIP in the Dorsal Horn of the Spinal Cord

The results of the behavioral testing showed that 14 days after L5 nerve injury, intraplantar injection of RTX in the ipsilateral paw significantly decreased the thermal hypersensitivity and the paw withdrawal latency reached the basal level compared with the right control paw. This effect continued for up to 28 days (Figure 1A). Therefore, we decided to examine the neurochemical changes 14 days after the intraplantar injection of RTX (0.002%, 100 µL) in the left hind paw.

**Figure 3:**
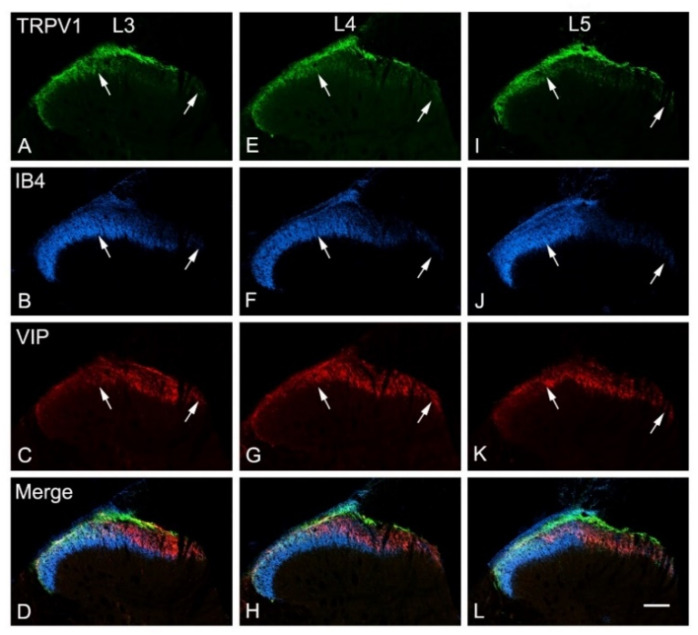
Confocal images of transverse sections of L3, L4 and L5 spinal segments showing the immunoreactivity of TRPV1, IB4 and VIP 14 days after intraplantar injection of RTX (0.002%) in the left hind paw. RTX treatment caused marked downregulation of TRPV1 (**A**,**E**,**I**), IB4 (**B**,**F**,**J**) in the medial aspect of the dorsal horn (between two white arrows) of L3–L5 segments, and in the same areas, VIP was upregulated (**C**,**G**,**K**). The area labelled between white arrows receives primary afferents originating from L3, L4 and L5 nerves, which supply the plantar skin in rats. (**D**,**H**,**L**) are merged images. Scale bar 100 µm.

**Figure 4:**
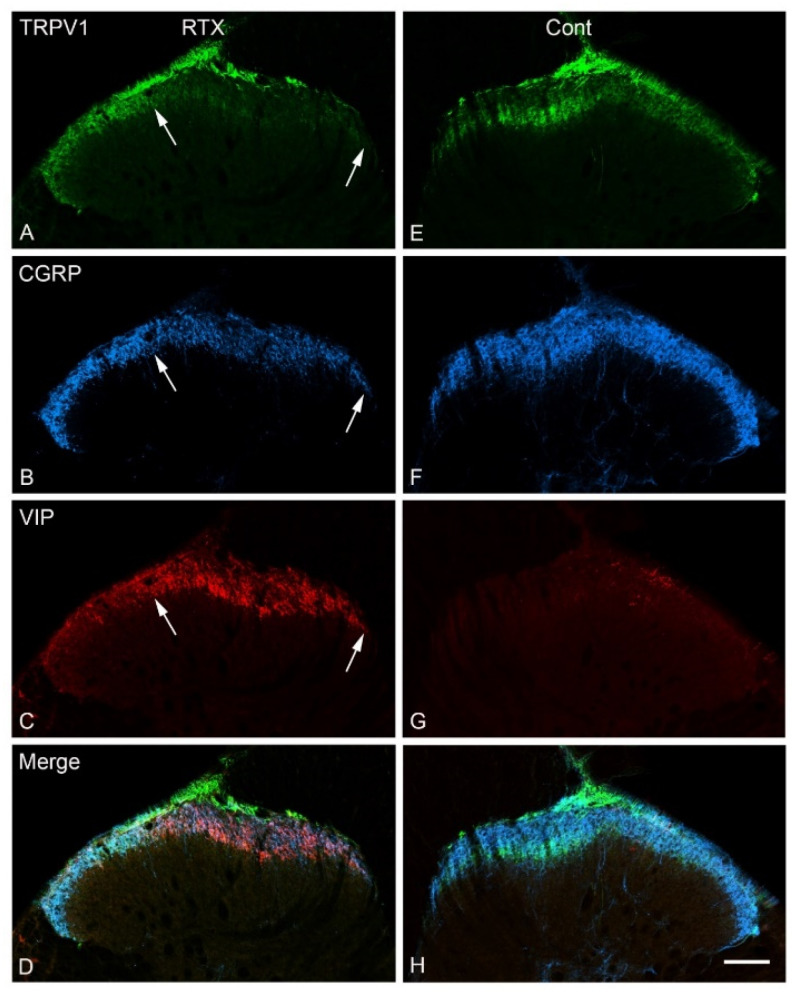
Confocal images of a transverse section of the L4 spinal segment showing TRPV1, CGRP and VIP immunoreactivity 14 days after intraplantar injection of RTX (0.002%) in the left hind paw. RTX treatment produced a marked downregulation of TRPV1-(**A**), CGRP-(**B**) and upregulation of VIP-immunoreactivity (**C**) in the same medial aspect of the superficial laminae of the spinal cord ipsilaterally (between two white arrows). No clear changes were observed in the contralateral (cont) side of the dorsal horn of the spinal cord (**E**–**H**). (**D**,**H**) are merged images. Scale bar = 100 µm.

**Figure 5:**
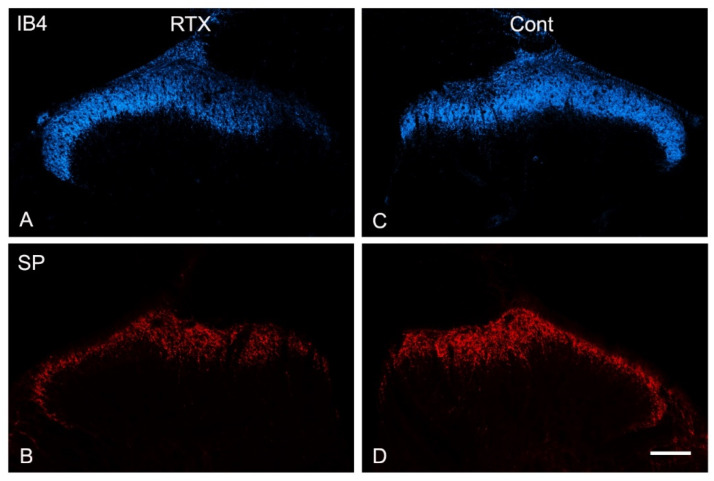
Representative confocal images of L4 spinal segment showing that intraplantar RTX treatment caused downregulation of IB4 (**A**) and SP (**B**) in the medial aspect of the dorsal horn compared with the normal expression of IB4 and SP in the contralateral (cont) side (**C**,**D**). Scale bar = 100 µm.

Triple and double immunofluorescent labelling using different sets of nociceptive markers were carried out in L3–L5 spinal cord segments. In the control contralateral side, profound immunoreactivities of TRPV1, IB4, CGRP and SP and only sparse immunoreactivity of VIP were localized in laminae I–II of the dorsal horn of the spinal cord (Figure 3, Figure 4 and Figure 5). Triple immunofluorescent labelling showed that the RTX injection caused downregulation of TRPV1, IB4 and CGRP in the same medial aspect of the dorsal horn of the ipsilateral side of the L3–L5 segments (Figure 3, Figure 4 and Figure 5). In contrast, RTX caused upregulation of VIP labelling in the same areas where TRPV1-, IB4- and CGRP-immunoreactivities were downregulated (Figure 3 and Figure 4). Similarly, RTX caused modest downregulation of SP in the L4 spinal dorsal horn in the same areas that showed downregulation of TRPV1 (Figure 5). 

The primary afferent fibers terminate and are somatotopically arranged in the dorsal horn of the spinal cord. Primary afferents from the limbs and the peripheral part of the body terminate in the medial part of the dorsal horn, while the primary afferents from the skin of the back terminate in the lateral part of the dorsal horn [38,39,40,41,42,43]. Accordingly, all neurochemical changes mentioned above following the intraplantar injection of RTX were observed in the medial part of the dorsal horn. Subsequently, a small area in the lateral aspect of the ipsilateral dorsal horn of the spinal cord showed neither downregulation of TRPV1, IB4, SP and CGRP nor upregulation of VIP (Figure 3A–C, Figure 4A–C and Figure 5A,B).

### 3.5. Effects of Intraplantar Injection of RTX on the Immunoreactivity of TRPV1, IB4, CGRP and VIP in the DRGs

**Figure 6:**
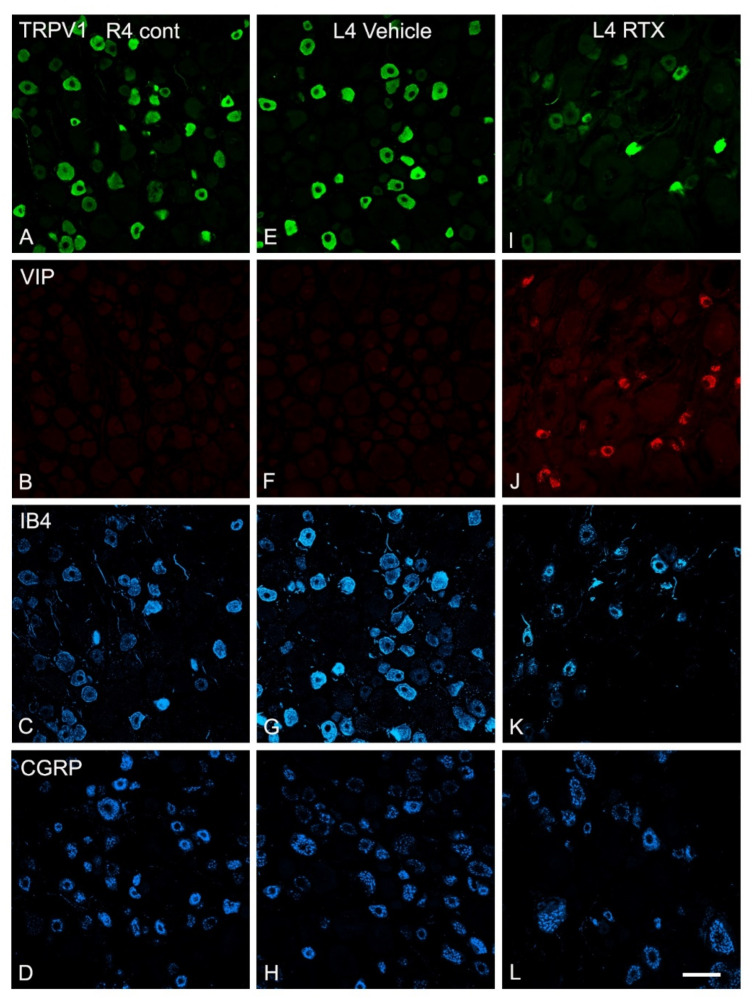
Triple immunofluorescent labelling for TRPV1, VIP, IB4 and CGRP in control right L4 DRG (R4 cont, **A**–**D**) and left L4 DRGs following 14 days of the intraplantar injection of vehicle (**E**–**H**) or 0.002% RTX (**I**–**L**) in the left hind paw. RTX treatment caused a remarkable reduction in the number of TRPV1+ neurons (**I**), IB4+ neurons (**K**) and CGRP+ neurons (**L**) in the L4 DRG compared to the right control (R4) and vehicle-treated L4 DRGs (**A**,**C**,**D**,**E**,**G**,**H**). In contrast, the number of VIP+ neurons were increased in the RTX-treated L4 DRG (**J**) compared to the right control (R4) or vehicle-treated L4 DRGs (**B** or **F**). Scale bar = 50 μm.

TRPV1, IB4 and CGRP were expressed in numerous numbers of small- and medium-sized neurons in the normal DRG. Triple immunofluorescent labelling showed that 14 days after RTX injection in the skin of the left hind paw, the expression of TRPV1-, CGRP- and IB4-immunoreactivity were downregulated in the L4 DRGs compared to vehicle-treated and right control L4 DRGs (Figure 6). Quantitative analysis showed that the percentage of TRPV1+ neurons was significantly reduced (*p* < 0.001; Table 3) in the L4 DRGs 14 days after RTX injection compared to vehicle-treated and right control L4 DRGs. Similarly, the percentage of IB4-positive (IB4+) and CGRP-positive (CGRP+) neurons in the L4 DRGs was also significantly reduced (*p* < 0.001; Table 3). 

In the normal DRG, only sparse labelled VIP+ neurons could be detected in the DRG. However, marked VIP upregulation was observed in the L4 DRG after 14 days of RTX treatment compared with only a few scattered VIP+ cells observed in the vehicle-treated and right control L4 DRGs (Figure 6).

**Figure 7:**
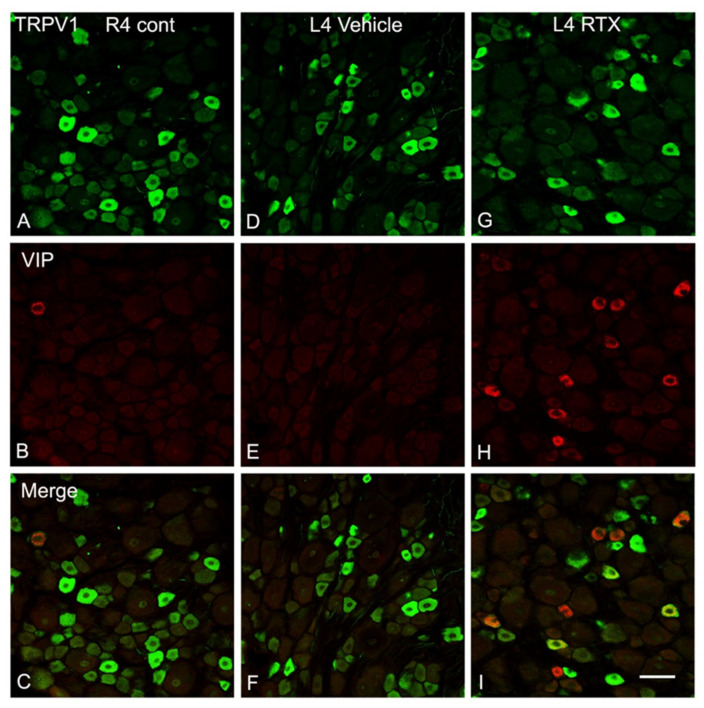
Double immunofluorescence staining of TRPV1 (green) and VIP (red) in the right control L4 (R4) DRG (**A**–**C**) and left vehicle (**D**–**F**) and RTX (**G**–**I**) L4 DRGs after 40 h of intraplantar injection of RTX/vehicle in the left hind paw. The number and intensity of TRPV1+ neurons in the L4 DRG of RTX-injected rats were significantly low compared to the right control and vehicle-injected L4 DRG. However, the number of VIP+ neurons was profoundly increased in the RTX-injected L4 DRG compared with a few scattered VIP+ neurons in the right control and vehicle-injected rats. Interestingly, most VIP+ neurons were colocalized with TRPV1+ neurons (**I**). Scale bar = 50 µm.

In a preliminary experiment, we found no clear VIP immunolabelling after 24 h, but marked upregulation was observed after 40 h of intraplantar RTX injection (Figure 7). For quantification, therefore, double immunofluorescent staining was used and TRPV1- and VIP-labelled neurons were counted 40 h and 14 days after intraplantar RTX injection. The results showed a remarkable loss of TRPV1+ neurons in the ipsilateral L4 DRG of RTX-treated rats compared to vehicle-treated and right control L4 DRGs. In contrast, the number of VIP+ neurons were markedly increased in the ipsilateral L4 DRG compared with few VIP+ neurons in the vehicle-treated and right control L4 DRGs. Most of the VIP+ cells (68%) after 40 h of RTX treatment were colocalized with TRPV1+ neurons in the ipsilateral L4 DRG, implying that RTX injection affected the TRPV1-expressing unmyelinated neurons. Further analysis showed no significant difference in the percentage of VIP+ neurons in the ipsilateral L4 DRGs after 40 h and 14 days of RTX injection (Table 4).

### 3.6. Effects of Intraplantar Injection of RTX on the mRNA and Protein Expression Level of TRPV1 in the DRG

**Figure 8:**
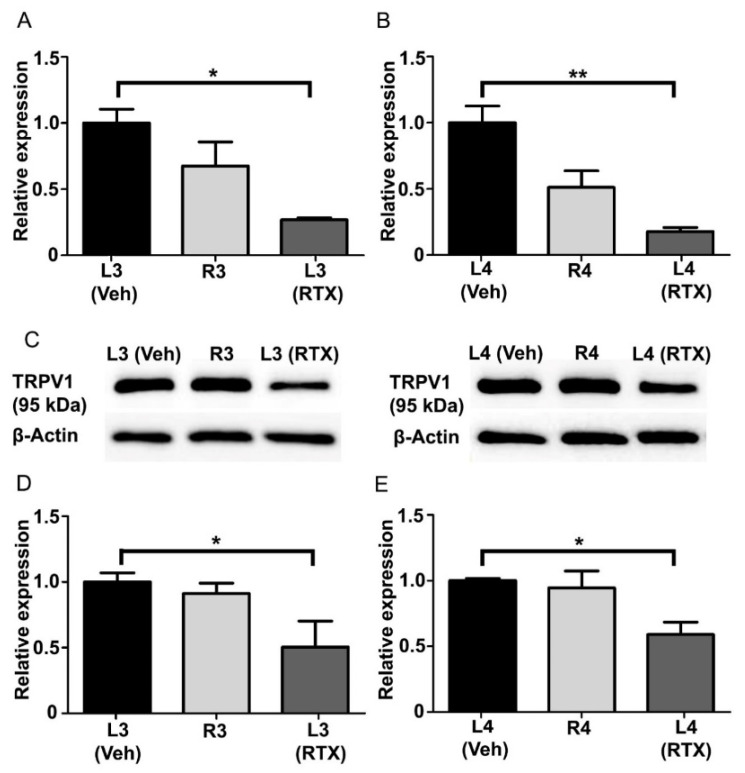
The level of TRPV1 mRNA and protein expression in left L3 and L4 DRGs after 14 days of intraplantar injection of RTX/vehicle in the left hind paw. A significant decrease in TRPV1 mRNA (**A**,**B**) and protein (**C**–**E**) expression was observed in the left L3 and L4 DRGs ipsilaterally injected with RTX compared to vehicle-injected or contralateral right control L3 and L4 DRGs. [* *p* < 0.05; ** *p* < 0.01, * vehicle vs. RTX-injected groups (*n* = 3–5 per group)].

After 14 days of RTX or vehicle injection in the left hind paw of rats, the mRNA and protein expression levels of TRPV1 were examined in the L3 and L4 DRGs using qRT-PCR and Western blot techniques, respectively. TRPV1 mRNA expression level was found significantly (Figure 8A,B; *p* < 0.05; *p* < 0.01) decreased in the ipsilateral RTX-treated left L3 and L4 DRGs compared to vehicle-treated L3 and L4 DRGs. We also observed no significant decrease in the expression level of TRPV1 mRNA in the right control R3 and R4 DRGs compared to vehicle-treated left L3 and L4 DRGs. However, TRPV1 protein expression level was significantly (Figure 8C–E; *p* < 0.05) decreased in the ipsilateral RTX-treated left L3 and L4 DRGs compared to vehicle-treated left L3 and L4 DRGs, as well as contralateral right control R3 and R4 DRGs.

### 3.7. Effects of Intraplantar Injection of RTX on the c-Fos Activation in the Spinal Cord 

**Figure 9:**
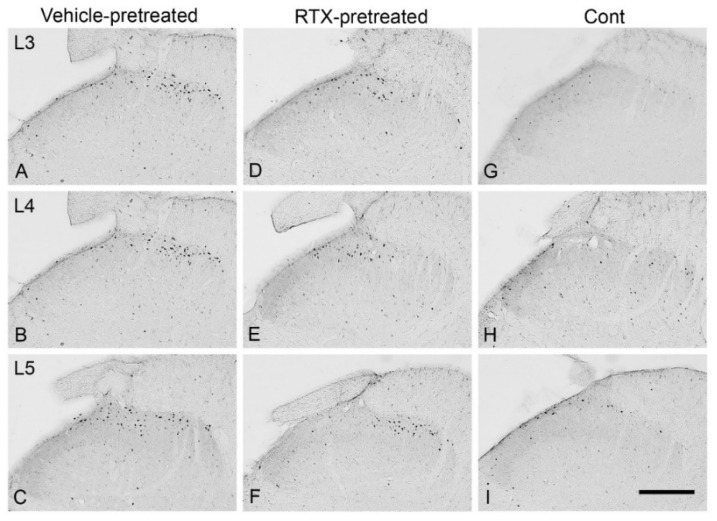
Images showing c-fos expression in sections of L3–L5 spinal segments 90 min after intraplantar injection of RTX (0.002%, 100 µL) in the left hind paw of rats that were administered the same amounts of RTX or vehicle injection 14 days earlier. A profound expression of c-fos was observed in the medial half areas of the L3 (**A**), L4 (**B**), and L5 (**C**) dorsal horn of the spinal cord of animals that were injected with vehicle 14 days earlier. In contrast, animals injected with RTX showed significant suppression of c-fos expression in the dorsal horn of L3 (**D**), L4 (**E**), L5 (**F**) of spinal segments. The right control sides of L3 (**G**), L4 (**H**), L5 (**I**) showed a basal level of c-fos expression (Scale bar = 200 μm).

**Figure 10:**
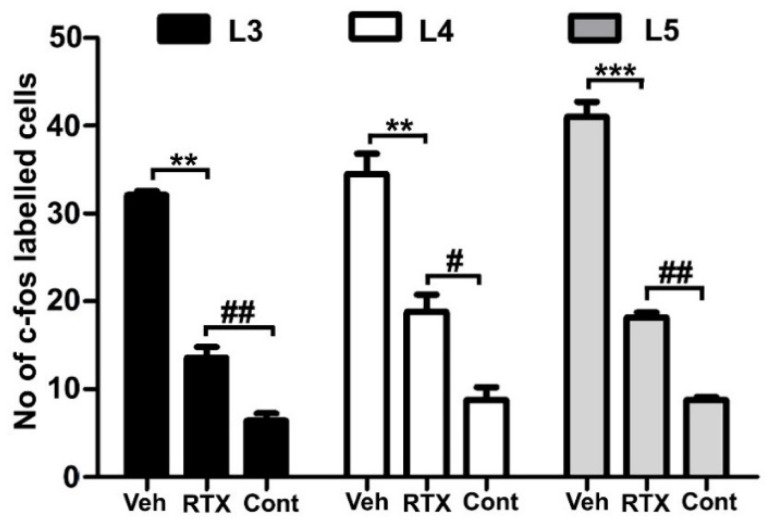
The quantification data showed a significant reduction (** *p* < 0.01; *** *p* < 0.001, One-way ANOVA) in the number of c-fos labelled neurons in the L3, L4 and L5 spinal segments of RTX-injected rats compared to the vehicle-injected rats. However, significantly more c-fos expression was observed in the RTX-injected rats compared with control right sides of L3, L4, and L5 segments [(# *p* < 0.05, ## *p* < 0.01) *n* = 3–4].

Intraplantar injection of 0.002% RTX-induced c-fos in the medial aspect of ipsilateral superficial layers (laminae I–II) of the dorsal horn of L3–L5 spinal segments in those animals that were administered vehicle treatment 14 days earlier (Figure 9). In comparison, the quantification data showed that the RTX-induced c-fos expression was significantly reduced in animals that were administered RTX 14 days earlier compared to vehicle-injected animals (*p* < 0.01; *p* < 0.001) (Figure 10), but still significantly more c-fos expression was exhibited compared with control right sides of L3, L4 and L5 segments (# *p* < 0.05, ## *p* < 0.01). Further analysis showed no significant difference in the number of c-fos labelled neurons in the contralateral side of both RTX- and vehicle-injected animals.

### 3.8. Effects of Intraplantar Injection of RTX on the Immunoreactivity of PGP 9.5 and CGRP in the Skin of the Hind Paw of Rats

**Figure 11:**
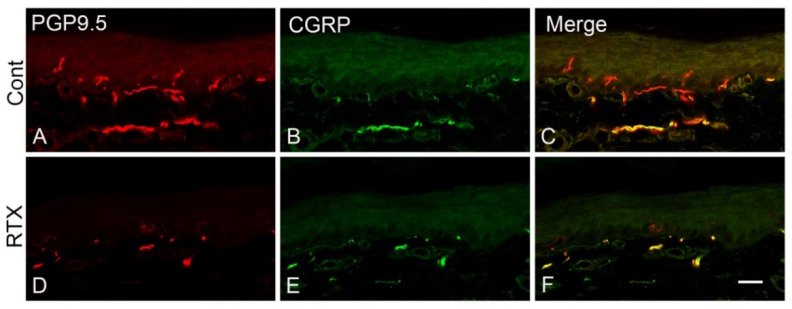
Effect of intraplantar injection of RTX (0.002%) in the hind paw on the distribution of PGP 9.5- and CGRP-labelled nerves in the injected skin. Panels (**A**–**C)** show normal PGP 9.5- and CGRP-labelled nerves in the vehicle-injected rats. RTX injection decreased the number of both PGP 9.5- and CGRP-labelled nerves (**D**–**F**). Scale bar 25 µm.

The plantar skin of the control and vehicle-injected animals showed normal nerve endings stained with PGP-9.5 and CGRP antibodies (Figure 11A-C). In comparison, both PGP 9.5- and CGRP-labelled nerve endings were decreased 14 days after the intraplantar RTX injection (Figure 11D–F).

## 4. Discussion

We determined the analgesic effects of intraplantar injection of RTX on the development and prevention of nerve-injury-induced neuropathic pain in rats. The dose and the volume of RTX were based on previous studies [11,27]. In our previous study, we used different doses of capsaicin and RTX and found 0.002% of RTX produced maximal effects when applied perineurally to alleviate neuropathic pain in the SNL model without neuronal and nerve fiber loss [11]. The same volume of 100 µL was used previously in rats for intraplantar injection [27,28]. Following RTX or vehicle injection, animals were measured for the paw withdrawal latency in response to thermal and mechanical stimuli from 3–28 days.

The main results of the study showed that (1) the intraplantar injection of RTX completely alleviates and prevents nerve-injury-induced thermal and mechanical hypersensitivity. (2) in addition to its known effects on the ablation of TRPV1 in the nerve terminals in the skin, intraplantar RTX injection has also produced a decrease in the levels of TRPV1, CGRP and IB4 in the corresponding DRGs and centrally in the dorsal horn of the spinal cord. (3) the decrease in the levels of TRPV1, CGRP and IB4 and upregulation of VIP were very likely due to neuroplastic changes in the primary sensory neurons rather than neuronal death or nerve degeneration and loss. (4) RTX-induced c-fos activation in the spinal neurons was suppressed in animals that were administered RTX injection 14 days earlier.

### 4.1. Neurochemical Changes Following Intraplantar Injection of RTX

Previous works in animals and humans showed that intraplantar injection of capsaicin or RTX caused the loss of labelled dermal and epidermal nerve fibers [37,44,45]. In these studies, the investigators visualized peripheral nerve fibers immunohistochemically using the PGP-9.5 antibody as a pan-neuronal marker [37,44]. In humans, a loss of PGP 9.5 immunoreactive fibers was observed in the epidermis with a moderate disruption of nerve fibers in the sub-epidermis after a low dose of capsaicin during the first 14 days after skin injection. Higher doses of capsaicin caused complete loss of PGP 9.5 immunoreactive fibers in the epidermis and subepidermal plexus [45] Reinnervation of the epidermis began during the third and fourth weeks after the capsaicin injection. In another human study when capsaicin cream was applied to the skin, PGP 9.5 labelled epidermal nerve fibers were greatly reduced, but 6 weeks after capsaicin discontinuation, the nerves reentered the epidermis [46]. It was suggested that the initial loss and subsequent reappearance of PGP 9.5 immunoreactivity coincided with degeneration and subsequent regeneration [45]. The reversibility of cutaneous denervation induced by topical treatment of RTX should be interpreted with caution as it depends on several factors, including the route, dose and number of injections.

The current study supports these findings and extends further by showing a reduction of TRPV1 and other nociceptive neuronal markers including CGRP, IB4 and SP in the primary sensory neurons that supply the injected plantar skin. However, it is controversial whether this loss of PGP-9.5- and CGRP-immunoreactivity in the skin could be due to nerve degeneration and loss or profound downregulation in their levels that rendered them immunohistochemically undetectable. The latter, in our opinion, is very likely the case for the following reasons. First, we recently investigated histologically the effects of perineural application of the same dose of RTX (0.002%) on the L4 nerve. The results showed no degeneration of the unmyelinated axons of the L4 and sciatic nerves (which is formed mainly by L4 and L5 nerves) or corresponding DRG neurons [11]. In addition, we found no difference between the sizes of unmyelinated nerve fibers in rats treated with RTX compared with control vehicle-treated rats [11]. Second, a similar previous study showed no significant damage to the unmyelinated nerve fibers following the perineural application of RTX on the sciatic nerve [47,48]. Third, decreased PGP-9.5 labelling of the cutaneous nerves after intraplantar injection, cream application and patch application of capsaicin was found to be reversible [19,45,46,49]. Fourth, further support from another study showed no difference in the transport of neuroanatomical tracer wheat germ agglutinin conjugated with horse-radish-peroxidase (WGA-HRP) when injected into the contralateral sciatic nerve compared with the ipsilateral nerve perineurally treated with capsaicin [50]. Fifth, it was reported that intraplantar injection of capsaicin prevented the skin-incision-induced changes in 99 of 126 genes in the L4–L6 DRGs in rats [51] and suppressed Ca^2+^ responses in the L5 DRG in mice [52].

### 4.2. Intraplantar RTX Injection Caused VIP Upregulation in the Primary Sensory Neurons

Normally, VIP immunoreactivity could not be detected in the DRG and only sparse labelling could be observed in the spinal cord, but it is upregulated in the DRG and central nerve terminals in the spinal dorsal horn after peripheral axotomy. Therefore, the VIP upregulation has been used as a marker for injured unmyelinated and possibly thin myelinated primary afferent neurons [31,36,53]. Recently, we have observed VIP upregulation in the corresponding DRG and spinal cord after the perineural application of RTX onto the L4 nerve [11]. Similarly, in this study, we found upregulation of VIP in the L4 DRG and the superficial layer of the L3, L4 and L5 dorsal horn in the same areas where TRPV1, IB4 and CGRP were downregulated. The VIP upregulation was detected in 14% of the total neurons in L4 DRG and most of them (68%) were also TRPV1 positive after 40 h of RTX injection. First, this result indicated that RTX caused VIP upregulation in neurons that supply the plantar skin and exerted its action through TRPV1 located on the peripheral nerve [44]. Not all VIP+ neurons were TRPV1+ after 40 h of RTX injection because the TRPV1 began to be downregulated to a level that could not be detected immunohistochemically, which resulted in the absence of colocalization in a number of neurons.

Second, RTX injection caused TRPV1 downregulation, as shown by a decreased expression of its protein and mRNA levels and a reduced number of TRPV1+ neurons from 24% after 40 h to 8% after 14 days. This led to the drop in colocalization of VIP with TRPV1 in the DRG from 68% to 3.4%. Third, there was no significant difference between the percentage of VIP+ neurons in the DRG after 40 h (14%) compared with 14 days (13%) of RTX injection. Taken together, these results indicated that neurons in which TRPV1 was downregulated were still viable and capable of producing VIP and, thus, providing evidence against neuronal loss in the DRG.

### 4.3. Perineural vs. Intraplantar RTX Application in Treating Nerve-Injury-Induced Neuropathic Pain

We assessed the effect of intraplantar injection of RTX on thermal and mechanical hyperalgesia/hypersensitivity following L5 nerve injury [3]. As we reported previously [11], hyperalgesia is a term used in human medicine, thus, in animal models, it is more appropriate to describe these events as “hypersensitivity”.

We showed that capsaicin and RTX application on the uninjured adjacent L4 nerve produced significant alleviation of both thermal and mechanical hypersensitivity [11,54]. However, to achieve the baseline pre-injury level of withdrawal threshold and completely abolish both the mechanical and thermal hypersensitivity, both L3 and L4 nerves needed to be treated with RTX [11]. The treatment of both L3 and L4 uninjured adjacent nerves was needed to alleviate the hypersensitivity because, anatomically, both these nerves supply the plantar skin in rats that was tested in this animal model of neuropathic pain. These results were supported by the previous studies in which perineural application of RTX on the sciatic nerve suppressed both thermal and mechanical hypersensitivities [47] produced by applying loose constrictive ligatures around the sciatic nerve [55] and inhibited inflammatory hyperalgesia induced by injection of carrageenan in the plantar skin of the hind paw [26].

Perineural application of RTX on both L3 and L4 nerves caused a significant reduction in thermal and mechanical hypersensitivity caused by left L5 nerve injury three days post-surgery with further improvement over 1–2 weeks. However, it took 3–4 weeks for withdrawal latency to show no significant difference from that of the control right paw [11]. In the present study, the same animal model was used to establish neuropathic pain after L5 nerve injury, intraplantar injection of RTX caused significant analgesic effects by abolishing the thermal sensitivity in three days, but the sensory responses were back to basal level after 14 days. These interesting differences suggest that the early effects of intraplantar RTX injection on thermal hypersensitivity might be due to localized effects in the skin and the long-term effects could be attributed to the central effects at the level of the DRG. In support of this, our previous study [11], as well as the present study, showed downregulation of TRPV1, CGRP and IB4 in the primary sensory neurons, which are all involved in the sensory pain pathway and correlate with decreases in both heat and mechanical sensations.

As for the mechanical sensation, neither the perineural [11] nor the intraplantar injection (this study, [27]) of RTX produce any significant effect on tactile sensation using von Frey filament. This supports the concept that these sensations are not mediated through TRPV1 and are very likely by mechanosensitive ion channels such as PIEZOs [56]. However, it was interesting to notice that both perineural [11,54] and intraplantar (this study) administration of RTX alleviated mechanical hypersensitivity suggesting that TRPV1 is involved in the mechanical hyperalgesia and its downregulation following RTX treatment helped in reducing the nerve-injury-induced hypersensitivity.

The analgesic action of RTX against thermal hypersensitivity correlates well with the reduction in heat receptor TRPV1 [8,9,20]. In addition, as we have previously suggested [11], the reversal of the mechanical hypersensitivity after RTX treatment might be due to its effects on the reduction of CGRP, IB4, SP, as well as somatostatin and other ion channel levels in the primary afferent neurons that are colocalized with TRPV1 neurons and that are known to be involved in the transmission of peripheral nociception.

### 4.4. Mechanisms of Analgesic Action of RTX

Capsaicin and its analogue RTX have been extensively used to investigate the pathophysiology and their analgesic effects in acute and chronic pain. The analgesic effects of capsaicin and RTX are based on the observations of their initial activation which leads to the excitation of the sensory neurons in the DRG and the spinal cord through TRPV1, followed by a refractory state in which neurons do not respond to various stimuli by a process described as “defunctionalization” [19]. Capsaicin and RTX act through their specific receptor TRPV1 on the unmyelinated and thinly myelinated nerve fibers that originate from small- and medium-sized neurons in the DRG [57]. This is supported by the localization of TRPV1 in the small–medium sized neurons in the DRG and its distribution in the superficial layers (laminae I–II) of the dorsal horn of the spinal cord [11,13,15,58,59], and on the nerve fibers as well as on the cutaneous nerves [44,49], our unpublished observation. Different routes of administration of capsaicin and RTX, including oral, intrathecal, intracisternal, perineural, ganglionic, intraarticular, intraplantar and nasal, have been used to investigate and treat acute and chronic pain in animals and humans due to nerve injury, inflammation, cancer and osteoarthritis [20,21,22]. One main advantage of using peripheral and topical application is to avoid potential therapeutic issues and general side effects. The topical application of capsaicin is used widely as a tool for producing, as well as treating, pain in both clinical and preclinical studies. Either a low dose of capsaicin cream (<1% applied 2–3 times per day for 6–8 weeks) or a higher dose (8% as a single application) have been used as topical treatments for neuropathic pain [19]. However, evidence for the effectiveness of topical capsaicin is equivocal. A Cochrane database review concluded that a low concentration of topical capsaicin fared no better than placebo creams [18]. Furthermore, a later systematic review [60] reported that high concentrations of topical capsaicin could be helpful for approximately 10% of neuropathic pain patients. Alternatively, a perineural application of either capsaicin or RTX has been shown to alleviate neuropathic pain manifestations [11,47,54]. One of the important issues here is translating these preclinical data to humans. In this situation, the administration of RTX perineurally or into DRG would require a specialized physician trained to inject the drug using ultrasound- or CT-guided equipment [61]. Instead, intraplantar injection of RTX can be considered in treating chronic pain as it can be easily performed in outpatient clinics without the need of sophisticated equipment or specialized personnel.

In this respect, the current study showed that focal injection of RTX into the plantar skin alleviated and prevented thermal and mechanical hypersensitivity following peripheral nerve injury. These results were supported by previous works in which RTX in the plantar skin blocked the formalin-induced nociceptive behavior and inflammation-induced hyperalgesia [62], and capsaicin injection in the facial skin attenuated mechanical hyperalgesia lasting for more than 14 days in chronic constriction injury of the infraorbital nerve model of neuropathic pain [63,64]. In addition, cutaneous injection of capsaicin has also been shown to attenuate the skin-incision-induced thermal and mechanical hypersensitivity [52].

In summary, peripheral injury of the L5 nerve often leads to neuropathic pain manifestations in rats in the ipsilateral foot. The main finding of this work did not only highlight the advantage of using focal RTX treatment over systemic treatment with analgesic agents but showed that peripheral intraplantar administration of RTX can completely reverse and prevent neuropathic pain manifestations. Peripheral RTX administration suppresses the activation of the peripheral nerves and reduces the TRPV1 heat receptors in the injected skin as well as in the DRG and centrally in the spinal cord where the nerves of the injected skin are terminated. The increase in the latencies of thermal pain and the reduction in thermal hypersensitivity after RTX treatment are well correlated with the downregulation of TRPV1 in the primary sensory neurons and the reduction of c-fos (neuronal activity marker) labelled nuclei in the spinal neurons compared with vehicle-treated animals.

In addition, RTX treatment causes the downregulation of other nociceptive markers, e.g., CGRP, IB4, SP and possibly somatostatin and a number of ion channels, which are colocalized with TRPV1 in the primary sensory neurons. This very likely explains how the RTX treatment reversed mechanical hypersensitivity following peripheral nerve injury. Peripheral RTX also blocks the activation of the immediate early gene expression of c-fos centrally in the spinal cord. The early analgesic effects of RTX correlate with its effects in the skin peripherally. However, the long-lasting analgesia can also be attributed to another factor(s). In support of this, [64] showed that capsaicin induced the depolymerization of axonal microtubules, which determined the extent of the analgesia.

## 5. Conclusions

The work highlights the importance of the intraplantar injection of RTX as a potential pharmacological strategy to completely alleviate and prevent the development of peripheral neuropathic pain in animals and humans.

## Figures and Tables

**Table 1:** Experimental Procedures.

Groups	Experimental Procedures			
	Neuropathic Pain		Immunohistochemistry/Western Blotting/qRT-PCR	*c-Fos* Immunohistochemistry
	Treatment	Prevention		
Plantar RTX injection (0.002%, 100 µL)	Left L5 nerve injury to induce neuropathic pain, and after 7 days, RTX was injected in the plantar skin of the left hind paw.	RTX was injected in the plantar skin of the left hind paw, and after 7 days, the left L5 nerve was injured to induce neuropathic pain	RTX was injected in the plantar skin of the left hind paw, and rats were sacrificed after 14 days.	RTX was injected in the plantar skin of the left hind paw, and after 14 days, a second injection of the same dose and volume of RTX was injected. The rats were sacrificed 90 min after the second injection.
Plantar vehicle injection (10% Tween 80, 10% EtOH, 80% normal saline, 100 µL)	Left L5 nerve injury to induce neuropathic pain, and after 7 days, vehicle was injected in the plantar skin of the left hind paw.	The vehicle was injected in the skin of the left hind paw, and after 7 days, left L5 nerve was injured to induce neuropathic pain	The vehicle was injected in the plantar skin of the left hind paw, and rats were sacrificed after 14 days	Vehicle was injected in plantar skin of the left hind paw, and after 14 days, RTX was injected in the same left hind paw. The rats were sacrificed 90 min after the second injection.
Right control	The right hind paw of RTX-injected animals served as control.		Right DRG and spinal cord served as control.	

**Table 2:** Primary antibodies used, sources and dilutions.

Antibody	Immunogen	Host	Catalogue/Source	Dilution
TRPV1	YTGSLKPEDAEVFKDSMVPGEK	Guinea Pig	GP14100/Neuromics, MN, USA	1:5000
IB4	Purified *Griffonia simplicifolia lectin I* whole molecule	Goat	AS2104/Vector Laboratories, Peterborough, UK	1:1000
CGRP	Synthetic peptide corresponding to a portion of rat α-calcitonin gene-related peptide (CGRP)	Rabbit	T4032/Peninsula Laboratories, San Carlos, CA, USA	1:10,000
CGRP	Synthetic peptide corresponding to Rat CGRP conjugated to Keyhole Limpet Haemocyanin (KLH).	Sheep	ab22560/Abcam, Waltham, MA, USA	1:2000
VIP	Pure Porcine VIP	Rabbit	Gift from Prof J. Allen	1:2000
SP	Synthetic peptide	Rabbit	T-4107 (IHC7451)/Peninsula Laboratories, San Carlos, CA, USA	1:5000
PGP 9.5	Purified whole human PGP9.5	Rabbit	RA95101, Ultraclone, Isle of Wight, UK	1:1000
c-Fos	KLH-conjugated linear peptide corresponding to 14 amino acids from the N-terminal region of human c-Fos	Rabbit	ABE457/Merck Millipore, MA, USA	1:5000

**Table 3:** The percentage distribution of nociceptive markers in the DRGs after 14 days of intraplantar injection of RTX (0.002%) or vehicle in the skin of the left hind paw of rats. A significant decrease in the number of TRPV1, CGRP and IB4 immunoreactive neurons were observed in the ipsilateral L4 DRG compared with L4 vehicle or control contralateral right L4 DRGs. No significant difference was observed in the number of these nociceptive markers between vehicle-injected L4 and control right L4 DRGs (*n* = 3). *** *p* < 0.001 comparison of L4 RTX vs. L4 vehicle or control right L4.

DRGs (*n* = 3)	TRPV1 (%)	CGRP (%)	IB4 (%)
Left L4 (RTX)	12.12 ± 0.81 ***	25.23 ± 1.24 ***	19.70 ± 1.16 ***
Left L4 (vehicle)	36.45 ± 0.70	43.14 ± 0.81	40.58 ± 1.70
Right L4 (control)	31.34 ± 1.58	39.69 ± 1.88	42.58 ± 1.00

**Table 4:** The percentage distribution of TRPV1 and VIP immunoreactive neurons in the DRGs after 40 h and 14 days of intraplantar injection of RTX (0.002%) or vehicle in the skin of the left hind paw of rats. The number of TRPV1-labelled neurons was significantly reduced while a significant increase in VIP-labelled neurons was observed in the ipsilateral L4 DRG compared with L4 vehicle or control contralateral right L4 DRGs, after 40 h and 14 days of intraplantar injection of RTX. However, no significant difference was observed in the number of VIP-labelled neurons in the animals 40 h and 14 days after RTX injection. ** *p* < 0.01 or *** *p* < 0.001 comparison of L4 RTX vs. L4 vehicle or control right L4.

DRGs	40 H (*n* = 3)		14 Days (*n* = 3)	
	TRPV1	VIP	TRPV1	VIP
Left L4 (RTX)	23.58% ± 1.61 **	14.38% ± 0.65 ***	8.19% ± 0.77 ***	12.86% ± 1.22 ***
Left L4 (Vehicle)	34.62% ± 0.41	0.53% ± 0.06	31.74% ± 0.43	0.86% ± 0.04
Right L4 (Control)	31.19% ± 1.28	3.05% ± 0.72	28.78% ± 0.62	4.37% ± 0.16

## Data Availability

The data of this study will be available upon reasonable request to the corresponding author.
